# Exposure to antipsychotics and risk of stroke: self controlled case series study

**DOI:** 10.1136/bmj.a1227

**Published:** 2008-08-28

**Authors:** Ian J Douglas, Liam Smeeth

**Affiliations:** 1 Department of Epidemiology and Population Health, London School of Hygiene and Tropical Medicine, London WC1E 7HT

## Abstract

**Objectives** To investigate the association between use of typical and atypical antipsychotic drugs and incidence of stroke in patients with and without dementia.

**Design** Self controlled case series.

**Setting** UK based electronic primary care records in the general practice research database (GPRD).

**Participants** All patients registered in the database with a recorded incident stroke and at least one prescription for any antipsychotic drug before the end of 2002: 6790 eligible participants were identified and included in the final analysis.

**Main outcome measures** Rate ratio for stroke in periods of time exposed to antipsychotics compared with unexposed periods.

**Results** Use of any antipsychotic drug was associated with a rate ratio for stroke of 1.73 (95% confidence interval 1.60 to 1.87): 1.69 (1.55 to 1.84) for typical antipsychotics and 2.32 (1.73 to 3.10) for atypical antipsychotics. In patients receiving any antipsychotic drug, the rate ratios were 3.50 (2.97 to 4.12) for those with dementia and 1.41 (1.29 to 1.55) for those without dementia.

**Conclusions** All antipsychotics are associated with an increased risk of stroke, and the risk might be higher in patients receiving atypical antipsychotics than those receiving typical antipsychotics. People with dementia seem to be at a higher risk of an associated stroke than people without dementia and use of antipsychotics should, when possible, be avoided in these patients.

## Introduction

Concerns about an increased risk of stroke associated with atypical antipsychotic drugs first arose from limited trial data in 2002. On the basis of these results, Health Canada and Janssen-Ortho (the manufacturer of risperidone) issued a warning to prescribers to highlight this issue.^1^ In 2004 the United Kingdom’s Committee on Safety of Medicines (CSM) recommended avoiding the use of atypical antipsychotic drugs among people with dementia,^2^ although this decision was criticised as being detrimental to care of patients.^3^ Whether the risk of stroke associated with antipsychotic drug use differs among people with and without dementia is not known. More recently the European Pharmacovigilance working party considered the results of published and unpublished observational studies.^4^ ^5^ They concluded that there was some weak evidence that antipsychotic drugs increase the risk of stroke but no clear evidence that this risk was greater with atypical versus conventional antipsychotic drugs.^6^ The report suggested that uncontrolled confounding might have affected findings to date. In particular, the underlying cardiovascular risk in people prescribed and not prescribed antipsychotic drugs differs in ways that are difficult to quantify and control for.^7^ ^8^

We used a within person case series design to assess the risk of stroke associated with antipsychotic drugs. Using this technique, we compared the incidence of stroke during periods exposed and not exposed to the drugs of interest within individual patients,^9^ thus eliminating confounding between individuals. We examined whether some or all of the previously observed increased risk of stroke associated with antipsychotic drug use could be attributable to confounding, whether the risk of stroke associated with typical and atypical antipsychotic drug use differs, and whether the risk of stroke associated with antipsychotic drug use is higher among people with dementia.

## Methods

### General practice research database

The GPRD (www.gprd.com) was established in 1987 and was originally called the value added medical systems (VAMP) research bank.^10^ It currently contains information from over six million patients registered at over 400 general practice surgeries in the UK.^11^ Continuous information is recorded for each patient, including a record of each consultation, any diagnoses made, all prescribed medicine, and basic demographic data. The geographical distribution and size of general practices represented in the database are largely representative of the population of England and Wales, and the individuals registered on the database are representative of the whole UK population in terms of age and sex.^12^ The data held are rigorously checked and regularly audited and have been successfully used to conduct over 500 peer reviewed published studies. The information obtained from the database is entirely anonymous.

### Selection of participants

Patients were selected from the population registered with the database before 2003. Eligible participants were all patients with a first ever incident diagnosis of stroke at least 12 months after initial registration with the database. To be included in the study patients had to have had the incident stroke on or before 31 December 2002 and also to have been prescribed at least one antipsychotic medicine before this date. Concerns regarding the possible cerebrovascular effects of antipsychotic drugs in elderly people began to emerge around this time and so we did not include information on events after this date to eliminate the subsequent effect of possible changes in prescribing patterns. Also, because of concerns over repeat coding of a single event within the database, we considered only the first ever record of a stroke as truly incident and excluded any subsequent record from the analysis.

### Self controlled case series analysis

The self controlled case series method is derived from the cohort method and relies on intraperson comparisons in a population of individuals who have both the outcome and exposure of interest. Rate ratios compare the rate of events during exposed periods of time with the rate during all other observed time periods.^9^ This method removes the potential confounding effect of characteristics that vary between individuals, such as frailty and risk factors for vascular disease.

For each participant, we identified and classified all prescriptions for antipsychotic drugs before the end of 2002. When possible, we calculated the expected length of exposure after each prescription using information on pack size and dosing frequency. From all prescription records for which this information was available, we calculated the median length of exposure and assumed this length for all prescriptions where missing information prevented actual calculation. Each individual’s observation time was then divided into exposure periods as follows: fully exposed periods covered by the expected length of exposure, followed by a sequence of five 35 day periods after treatment up to a maximum of 175 days after the expected end of a treatment period. We included periods after treatment as we cannot be sure when treatment stopped, and these periods will represent a gradual shift from full exposure, to a washout period, and finally to an entirely unexposed state. All other periods of time were classified as baseline (unexposed). The figure[Fig fig1] shows how we classified the follow-up time for an individual participant with respect to exposure. We also searched each patient’s medical record for diagnoses indicating dementia before stroke to allow subgroup analysis in these patients.

**Figure fig1:**
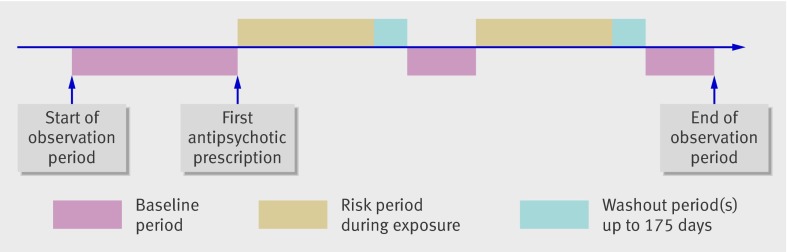
Pictorial representation of study design. Figure illustrates single individual prescribed antipsychotic drug during their observation period. All participants included in analysis had at least one prescription for an antipsychotic drug and a single incident stroke. Rate ratios presented are pooled estimates derived from rate of events during risk (exposed) periods divided by rate of events during baseline periods; age is adjusted for at all stages of analysis. Incident stroke can occur during any one of three exposure periods: baseline, exposed, or washout period

We estimated the relative rate ratios using conditional Poisson regression with Stata software, version 9 (StataCorp, College Station, TX), adjusting for age at stroke in five year bands. We assessed the impact of exposure to any antipsychotic medication, looked at the effect of any antipsychotic drug among patients with and without dementia, and measured the differential effects of typical and atypical antipsychotics among all patients and stratified by dementia status.

## Results

We identified 6790 eligible patients in the database with at least one prescription for an antipsychotic drug and a recorded incident stroke between January 1988 and the end of 2002. Of these, 905 patients were prescribed at least one atypical antipsychotic drug and 6334 were prescribed at least one typical antipsychotic drug during the study period. Table 1 shows the number of patients given a prescription for the most commonly prescribed antipsychotics in this population. The most commonly used typical antipsychotics were phenothiazines (5153 patients), and the most common atypical antipsychotic drug was risperidone (729 patients). Some 2290 (34%) patients had a single exposure period, 2406 (35%) had between two and five exposure periods, and the 2094 (31%) remaining had six or more. Table 2 gives demographic details of patients included in the study, of whom 4353 (64%) were women. The median age at first exposure to any antipsychotic drug was 80, while median age at the time of first recorded stroke was 81. Table 2 also shows details of each analysis subgroup; 1423 patients had a recorded diagnosis of dementia before the incident stroke and these patients were slightly older than those without dementia at the time the first antipsychotic drug was prescribed. In total, 5885 patients received a prescription for a typical but not an atypical antipsychotic drug during the study period, and 456 patients received prescriptions only for atypical antipsychotics. The age at first recorded antipsychotic drug exposure was similar for patients exposed only to typical or atypical antipsychotics. The 449 remaining patients received prescriptions for both typical and atypical antipsychotics. Among patients with dementia, 1212 received only typical antipsychotics and 85 received only atypical antipsychotics. The median duration of total observation period included in the analysis was at least four years for each subgroup.

**Table 1 tbl1:** Summary of antipsychotic drug prescribing in study population

	No (%) of patients with at least one prescription
**Atypical antipsychotics**	
No of patients	905*
Risperidone	729 (81)
Olanzapine	165 (18)
Amisulpride	39 (4)
Quetiapine	38 (4)
**Typical antipsychotics**	
No of patients	6334*
Phenothiazine	5153 (81)
Butyrophenone (haloperidol and benperidol)	1243 (20)
Thioxanthine (flupentixol and zuclopenthixol)	754 (12)
Sulpiride	160 (3)

*Individuals might have been prescribed >1 drug from each category and therefore appear in >1 row. Patients might also have been prescribed both typical and atypical antipsychotics.

**Table 2 tbl2:** Demographic details of study populations. Figures are numbers of patients and medians (interquartile ranges)

Patient group (n=6790)	First exposure		Duration of available data (years)	Age at first exposure (years)	Age at time of stroke (years)	Duration of antipsychotic exposure (years)
	Before stroke	After stroke				
Any antipsychotic drug	4336	2454	6.1 (3.1-9.8)	80.0 (72.7-86.1)	80.7 (73.0-86.8)	0.37 (0.10-1.23)
Dementia before stroke	1276	147	4.1 (2.1-7.5)	82.1 (76.8-87.1)	83.7 (78.6-88.5)	0.68 (0.22-1.55)
No dementia before stroke	3060	2307	6.7 (3.6-10.2)	79.2 (71.1-85.8)	79.3 (71.6-86.2)	0.30 (0.08-1.11)
Typical antipsychotic drug only	3845	2040	5.9 (3.1-9.3)	80.1 (72.6-86.2)	80.9 (73.4-87.0)	0.33 (0.08-1.18)
Atypical antipsychotic drug only	179	277	7.2 (3.0-11.1)	80.3 (73.8-86.4)	79.6 (72.6-85.3)	0.29 (0.09-0.76)
Dementia before stroke + typical antipsychotic drug only	1092	120	4.0 (2.1-7.0)	82.5 (77.0-87.2)	83.9 (78.8-88.7)	0.64 (0.21-1.51)
Dementia before stroke + atypical antipsychotic drug only	67	18	4.9 (1.8-10.9)	80.6 (76.3-87.7)	80.8 (76.4-87.9)	0.46 (0.19-0.96)
No dementia before stroke + typical antipsychotic drug only	2753	1920	6.4 (3.5-9.9)	79.2 (70.9-85.8)	79.8 (71.7-86.3)	0.26 (0.08-1.04)
No dementia before stroke + atypical antipsychotic drug only	112	259	7.8 (4.0-11.2)	80.3 (73.3-86.2)	78.9 (71.6-84.6)	0.23 (0.08-0.71)

The rate ratio for stroke among all patients prescribed any antipsychotic drug was 1.73 (95% confidence interval 1.60 to 1.87), comparing exposed with unexposed baseline periods (table 3). During the periods after treatment the rate ratio fell towards unity. During exposed periods, the rate ratio was 1.69 (1.55 to 1.84) for patients receiving only typical antipsychotics and 2.32 (1.73 to 3.10) for patients receiving only atypical antipsychotics. During periods of treatment with any antipsychotic drug the rate ratio was 3.50 (2.97 to 4.12) in patients with recorded dementia before stroke and 1.41 (1.29 to 1.55) in patients with no record of dementia before stroke. In patients with dementia and only typical antipsychotic drug prescriptions, the rate ratio for stroke was 3.26 (2.73 to 3.89). This compares with a figure of 5.86 (3.01 to 11.38) in patients with dementia and only treated with atypical antipsychotics. Patients without dementia before stroke and receiving only typical antipsychotics had a rate ratio of 1.40 (1.26 to 1.54) compared with a figure of 1.90 (1.36 to 2.65) in patients without dementia and receiving only atypical antipsychotics. In all analysis subgroups, the rate ratio for stroke subsequently fell towards unity during the phase after treatment.

**Table 3 tbl3:** Case series analysis for antipsychotic drugs: association between exposure and stroke. Figures are rate ratios (95% confidence intervals)

	Any antipsychotic drug	Typical only†	Atypical only†
**All patients (n=6790) **			
No on group	6790	5885	456
Exposed *v* unexposed periods	1.73 (1.60 to 1.87)	1.69 (1.55 to 1.84)	2.32 (1.73 to 3.10)
Days after treatment:			
1-35	1.73 (1.56 to 1.91)	1.69 (1.52 to 1.89)	1.81 (1.15 to 2.85)
36-70	1.66 (1.44 to 1.90)	1.63 (1.41 to 1.89)	2.22 (1.23 to 4.03)
71-105	1.61 (1.37 to 1.90)	1.57 (1.32 to 1.87)	1.29 (0.52 to 3.23)
106-140	1.23 (1.01 to 1.51)	1.27 (1.03 to 1.56)	0.32 (0.05 to 2.34)
141-175	1.06 (0.84 to 1.33)	1.06 (0.83 to 1.35)	0.76 (0.19 to 3.13)
**Patients with recorded dementia (n=1423)**			
No in group	1423	1208	85
Exposed *v* unexposed periods	3.50 (2.97 to 4.12)	3.26 (2.73 to 3.89)	5.86 (3.01 to 11.38)
Days after treatment:			
1-35	4.03 (3.34 to 4.87)	3.74 (3.05 to 4.59)	5.70 (2.50 to 12.98)
36-70	3.04 (2.33 to 3.96)	2.92 (2.20 to 3.88)	4.41 (1.40 to 13.89)
71-105	2.71 (1.97 to 3.73)	2.40 (1.69 to 3.41)	3.50 (0.76 to 16.25)
106-140	2.14 (1.45 to 3.15)	2.16 (1.44 to 3.23)	2.21 (0.28 to 17.34)
141-175	1.53 (0.95 to 2.44)	1.49 (0.90 to 2.44)	2.40 (0.30 to 18.88)
**Patients without recorded dementia (n=5367)**			
No in group	5367	4673	371
Exposed *v* unexposed periods	1.41 (1.29 to 1.55)	1.40 (1.26 to 1.54)	1.90 (1.36 to 2.65)
Days after treatment:			
1-35	1.30 (1.14 to 1.47)	1.30 (1.14 to 1.49)	1.28 (0.72 to 2.30)
36-70	1.42 (1.20 to 1.67)	1.41 (1.18 to 1.67)	2.04 (1.01 to 4.11)
71-105	1.43 (1.18 to 1.73)	1.44 (1.18 to 1.76)	1.02 (0.32 to 3.25)
106-140	1.08 (0.85 to 1.37)	1.11 (0.87 to 1.43)	NA
141-175	0.98 (0.75 to 1.28)	0.99 (0.75 to 1.30)	0.50 (0.07 to 3.62)

NA=not applicable (no events).

†Patients who received both typical and atypical antipsychotics not included in this analysis.

## Discussion

The previously observed increased risk of stroke associated with use of antipsychotic drugs is not attributable to differences in baseline cardiovascular risk between people prescribed and not prescribed these drugs. The risk of stroke is slightly higher with use of atypical rather than typical antipsychotic drugs. The magnitude of the increased risk of stroke associated with antipsychotic drug use is more than twice as great among people with dementia compared with those without.

### Strengths and weaknesses

A key advantage of this study is the use of the self controlled case series design, which is much less prone to problems of confounding than traditional case-control or cohort designs. Differences between patients are of little relevance as the risk comparisons are made entirely within patients. Also, the self controlled case series is more efficient than other observational study designs and so more precise estimates of effects can be made. We chose to censor follow-up for all patients at the end of 2002 as this was when concerns about the effects of antipsychotic drugs in patients with dementia first emerged. This should avoid possible biases arising from altered prescribing habits in the light of these findings.

Other main strengths of the study were that it was large and statistically powerful. Our study was based on routine clinical data from the UK general practice research database, which is largely representative of the population of the UK and so the results are likely to be highly generalisable. A potential weakness might relate to the quality of the clinical data. Drug prescriptions in the database are generated by practice computers to ensure the accuracy of the electronic prescribing records. Prescription data were highly detailed and recorded before people developed stroke so there was no potential for recall bias. Some patients were probably not taking their prescribed antipsychotics during periods we classified as exposed, though this would result only in a reduced effect estimate for antipsychotic drug exposure. There might have been some inaccuracy in our estimation of exposed and unexposed periods. We addressed this issue by measuring the relative risk of stroke during sequential periods after treatment, and, reassuringly, during these periods the relative risk fell consistently towards unity. Our analysis also suggests that the risk of stroke might not fall to baseline until six months after a patient stops taking an antipsychotic drug.

The validity of the clinical data included in the database has repeatedly been shown to be high.^13^ The rates for recorded stroke are comparable with estimates obtained from other epidemiological studies.^14^ The database has been widely used to study the epidemiology of stroke, with over 15 published papers (such as those by Smeeth et al,^15^ Mulnier et al,^16^ and Andersohn^17^), including the replication of results from two large randomised trials.^18^ ^19^ We chose not to include less serious outcomes related to stroke, such as transient ischaemic attack, as the ascertainment rate and accuracy of dating for these events is probably less reliable. Over 85% of the strokes identified in this study were recorded with codes that did not specify the subtype of stroke. Nevertheless, we carried out a subgroup analysis in the 233 patients with designated haemorrhagic stroke. The rate ratio for this subgroup, considering exposure to any type of antipsychotic drug, was 1.14 (0.71 to 1.84), indicating that the effect might be limited to non-haemorrhagic stroke.

We were unable to investigate possible mechanisms by which antipsychotics might cause stroke or why the risk seems to be greater with atypical antipsychotics. It is unclear if the effect represents a direct vascular toxicity or whether stroke might be a consequence of other well established side effects such as weight gain. To further investigate weight gain as a possible mechanism, we conducted a subgroup analysis censoring follow-up for each patient after the first three months of exposure to any antipsychotic drug, as weight gain is unlikely to substantially alter the risk of stroke within such a short timeframe. The resultant rate ratio was 2.66 (2.34 to 3.02), suggesting the effect is present shortly after a patient starts taking an antipsychotic drug. More information on a possible mechanism would be useful as further insight could facilitate effective minimisation of risk. Another possible limitation of the study design is that time varying confounders can be difficult to take into account. Initiation of an antipsychotic drug might also be associated with a change in exposure status for some other independent risk factor for stroke, such as a change in tobacco or alcohol consumption. While we have successfully controlled for age, data within the database do not generally allow precise measurements of subtle changes in behaviour patterns such as smoking and drinking.

### Results in context

Our results on the association between atypical antipsychotics and stroke are generally consistent with the results of previous placebo controlled clinical trials and observational studies, suggesting residual confounding is unlikely to be a major problem.^1^ ^2^ ^4^ ^5^ ^6^ ^20^ While few patients with dementia received atypical antipsychotics in our study and power is therefore reduced, the point estimate for the effect of atypical antipsychotics in this group is nearly 6, while the estimate from the risperidone clinical trial meta-analysis in such patients was 3.32 (1.43 to 7.70).^20^ Our estimate has wide confidence intervals, ranging from 3.01 to 11.38 and is therefore consistent with the effect seen in clinical trials. It is also consistent, however, with a more pronounced effect, which could reflect a genuinely different effect of atypical antipsychotics under less well controlled conditions than those attained in clinical trials. Before our study, the risk of stroke associated with typical antipsychotics was not clear. For example, the case-control study conducted by the UK’s Medicines and Healthcare Products Regulatory Agency (MHRA) suggested a stronger effect of risperidone on stroke than other antipsychotics^20^ but did not compare the effect of typical with atypical antipsychotics. We have established that all types of antipsychotics carry an increased risk, although the risk might be somewhat higher with the atypical drugs.

As the background risk of stroke in elderly patients is relatively high,^21^ we reaffirm that the risks associated with antipsychotic drug use in patients with dementia generally outweigh the potential benefits, and use of antipsychotic drugs in these patients should be avoided whenever possible. When the use of antipsychotic drugs is deemed necessary, our results indicate that typical antipsychotics are preferable to atypical antipsychotics with respect to serious cerebrovascular outcomes. By contrast, our results suggest a much more modest association between antipsychotics and stroke in patients without dementia, regardless of age (1.41, 1.29 to 1.55). This implies that the use of antipsychotics might be acceptable in elderly patients without dementia, although as with all treatment choices, a wider consideration of all potential risks and benefits would need to be taken into account.

**What is already known on this topic**

Atypical antipsychotics might increase the risk of stroke in elderly patients, but this association could be due to unmeasured or uncontrolled confounding
The extent to which typical antipsychotics are associated with an increased risk of stroke and whether the effect is the same in patients with dementia as those without is not known

**What this study adds**

Both typical and atypical antipsychotic drugs are associated with an increased risk of stroke and this association is unlikely to be caused by confounding
The risk of stroke in patients receiving antipsychotics seems to be greater in those with dementia than those without

## Notes

Ethical approval: Independent scientific advisory group of the general practice research database and the London School of Hygiene and Tropical Medicine ethics committee.

Provenance and peer review: Not commissioned; externally peer reviewed.

## Notes

**Cite this as:** *BMJ* 2008;337:a1227
